# CircRNA_400029 promotes the aggressive behaviors of cervical cancer by regulation of miR-1285-3p/TLN1 axis

**DOI:** 10.7150/jca.61437

**Published:** 2022-01-01

**Authors:** Yue Ma, Jing Liu, Zhuo Yang, Peng Chen, Dan-bo Wang

**Affiliations:** Department of Gynecology, Cancer Hospital of China Medical University, Liaoning Cancer Hospital and Institute, Shenyang, Liaoning 110042, P. R. China.

**Keywords:** Cervical cancer, circRNA_400029, miR-1285-3p, TLN1, proliferation, invasion

## Abstract

**Background:** Cervical cancer (CC) is the highest incidence of female malignant tumor in China. Circular RNAs (circRNAs) has been reported to affect CC progression by altering mRNA stability at the transcriptional level or binding to miRNAs to produce competitive endogenous RNA (ceRNA). For this purpose, our study was aimed to investigate the function effects and the potential regulatory mechanism of the circRNA_400029 in CC cells.

**Materials and methods:** The expression levels of circRNA_400029 and miR-1285-3p were detected by real time polymerase chain reaction (RT-PCR). Similarly, the mRNA and protein levels of TLN1 (Talin 1) was detected by RT-PCR and Western blot. Cell-Counting Kit-8 (CCK-8), EdU and Flow cytometry assay were used to detect cell proliferation, cell cycle and apoptosis. Then the Transwell assays were used to test cell migration and invasion. Besides this, the functional targets were confirmed by Dual luciferase reporter assays. Tumor xenograft in nude mice checked the result *in vivo*.

**Results:** To begin with, circRNA_400029 was upregulated in CC cells and tissue. Knockdown circRNA_400029 inhibited cell proliferation, migration and invasion while induced cell apoptosis. Interestingly, miR-1285-3p targeted circRNA_400029 and down-regulated of miR-1285-3p could reverse the effects of circRNA_400029 weak-expression on progression and apoptosis of CC cells. Moreover, TLN1 was up-regulated in CC cells and identified as a direct target of miR-1285-3p. Meanwhile, we found that miR-1285-3p negatively regulated the function of TLN1. Finally, the circRNA_400029/miR-1285-3p/TLN1 axis could affect tumor growth* in vivo.*

**Conclusion:** The overexpressed circRNA_400029 promoted CC proliferation, migration and invasion while deduced apoptosis by sponging miR-1285-3p to regulate TLN1. CircRNA_400029 was a potential onco-circRNA in CC, and might be a promised therapy target.

## Introduction

Cervical cancer (CC) is the most common malignant tumor in women with relatively high morbidity and mortality and remains the main cause of cancer-related deaths among women in developing countries 1. Prevention and control of CC is difficult on account of the risk factors such as bacterial factors, virus factors, environmental factors and genetic susceptibility 2. Among them, high risk human papillomavirus (HPV) infection is the main cause of CC 3. However, the occurrence and development of cervical cancer is a multi-stage, involving the abnormal regulation of multiple genes in the pathophysiological process. As screening has become more widespread and treatment has improved, the mortality rate for early-stage patients has been falling 4. However, the prognosis for patients with advanced stage is still poor.

Non-coding RNAs, including long non-coding RNAs (lncRNAs), circular RNAs (circRNAs) and microRNAs (miRNAs), do not encode proteins but interact with other RNAs, proteins and DNA to modulate their functions have become the stars of molecular biology research 5. Recent evidence suggested that circRNA acted as putative biomarkers involved in the pathogenesis of human cancer by regulating different gene functions 6-8. CircRNAs is a special structure of non-coding RNA with covalently closed circular structure, which is produced by the tail-head splicing of its host gene pre-messenger RNA (mRNA). It is widely expressed in eukaryotes and plays a role in specific patterns of tissue and developmental stages. The circRNA circFoxO3a was identified as a serum biomarker for squamous cervical cancer (SCC) 9. Furthermore, a previous document uncovered that the upregulation of circRNA_PVT1 could induce invasion and metastasisi of cervical cancer cells via exosome pathway 10. Meanwhile, knockdown of circ-0084927 restrained proliferation and facilitated apoptosis through sponging miR-634 and regulating TPD52 level in CC 11. These outcomes showed that circRNA had a great effect on CC and could serve as a potential clinical test biomarker in CC treatment.

MicroRNAs (miRNAs) are noncoding RNA molecules containing approximately 23 nts that playing gene-regulatory roles on proteins expression and evolution in different cells 12. Previous reports presented that miR-7 modulated multiple molecular targets and served as a potential therapeutic target in CC 13. Up-regulated miR-216a-3p could suppress the proliferation and invasion of CC through modulated multiple molecular targets and served as a new positive regulatory factor of YAP signaling pathway. miRNA-499a promotede the cell migration and invasion and enhanced chemoresistance of CC cells by targeting SOX6 14. In addition, exosomal miRNA is also an important target for remodeling tumor microenvironment and molecular diagnosis 15.

In this study, over-expressed circRNAs in CC were obtained through high-throughput microarray, and the potential molecular targets were analyzed and verified. We found the circRNA_400029 was overexpressed in CC cells and tissue. It contributed the CC cell proliferation, migration and invasion while retarded apoptosis. Mechanically, it was achieved by the circRNA_400029/miR-1285-3p/TLN1 (Talin 1) axis.

## Materials and methods

### Cell culture and cell transfection

Four CC cell lines (SiHa, HeLa, CaSki, C-33A) and human cervical epithelial immortalized cell line H8 were obtained from were obtained from China Medical University (Shenyang, China). They were cultured with DMEM (Gibco BRL, USA) and 10% fetal bovine serum (FBS, USA) placed to incubator with 5% CO2 and 37 °C.

All oligomers used in this study including siRNA targeting circRNA_400029 junction site, miR-1285-3p mimics and inhibitors were commercially obtained from Sangon Biotech (Shanghai, China). Before transfection the SiHa and CaSki were cultured in 24-well plates with a density of 30% for 24 h, siRNAs were transfected into SiHa and CaSki cells by Lipofectamine 2000 (Invitrogen, USA). After transfection and cultured for 24 h, 48 h and 72 h, samples were collected for the next experiment.

### Patients and specimens

A total of 50 CC tissues and their neighboring healthy tissues were obtained from Liaoning Province Cancer Hospital &Institute. All patients had a clear pathological diagnosis. The degrees of these CC patients were divided into stage on the basis of the AJCC TNM staging system. All patients had not received any therapy. All enrolled patients had no other tumors or systematic diseases shortening survival. These specimens were stored in -80 °C. And we got the approval of the Ethics Committee of Liaoning Province Cancer Hospital &Institute in this study and written informed consent was signed by every participant. The information of the patients were shown in Table 1.

### RNA isolation and RT-PCR

The total RNA of samples was separated and extracted by TRIzol (Invitrogen, USA). cDNA was obtained by the RevertAidTM First Strand cDNA Synthesis Kit (Fermentas, Canada). PCR was running in an ABI SYBR Green Master Mix (Invitrogen, USA). The primers sequences were as follows: 5'-CACAGTGCTGGGATTACA-3' and 5'-CAGGAGAATCGCTTGAATC-3' for circRNA_400029; 5'- TCTGGGCAACAAAGTGAG-3' and 5'- CTCAACTGGTGTCGTGGA -3' for miR-1285-3p; 5'-CTCGCTTCGGCAGCACA-3' and 5'- AACGCTTCACGAATTTGCGT-3' for U6; TLN-1: 5'- TGTAGAGGAGCACGAGACGC -3' and 5'- AAGGAGACAGGGTGGGAGC -3' for TLN1. These experiments were repeated 3 times and data was analyzed with 2^-ΔΔCt^ method.

### Nuclear and cytoplasmic separation assay

Following to the manufacturer's protocol, the PARIS kit (AM1921; Thermo Fisher Scientifc, Yokohama, Japan) divide the total cellular fractions into nuclear and cytoplasmic fractions. The kit isolates the nuclear and cytoplasmic components prior to RNA extraction, and ensures that RNA is isolated from the same experimental sample.

### Immunofluorescence

At room temperature, fix the cells with 4% paraformaldehyde PBS for 10 minutes, and infiltrate with 0.1% Triton-X and 1% BSA PBS for 30 minutes. DNA staining was performed using 4',6-diamino-2-phenylindole (DAPI). An ECLIPSE Ni microscope (Nikon, Japan) was used for imaging.

### RNA fluorescence *in situ* hybridization

SiHa and CaSki cells were fixed with 4% formaldehyde for 15 min, and then washed with PBS, treated with pepsin and dehydrated with ethanol. RNA fluorescence *in situ* hybridization (FISH) was performed using a Ribo^TM^ Fluorescent *In situ* Hybridization Kit (RiboBio, China) according to the manufacturer's instructions. The FITC-labeled hsa circRNA_400029 probe were detected and observed by ECLIPSE Ni microscope (Nikon, Japan).

### CCK-8 proliferation vitality assay

The cell proliferation viability were detected by the Cell-Counting Kit-8 (Dojindo Laboratories). SiHa and CaSki cells were cultivated into a 96-well plate with a density of 5×10^4^ cells/well and incubated in 37 °C for 24, 48, 72 or 96 hours. Then the cells proliferation viability was detected by a microplate reader (Bio-Rad, CA) using spectrophotometry.

### Transwell assay

Cells migration and invasion were detected by transwell cell culture chambers with or without Matrigel (Corning Life Sciences, USA). To begin with, cells were added 100 ml of no-serum medium and inoculated to the upper chamber, and 500 ml of DMEM with 10% serum was inoculated to the lower chamber. Then the cells attaching to the lower surface of the upper chamber was disposed with 4% paraformaldehyde (PFA) after 24 h of incubation. Observation under the microscope after staining with crystal violet and statistics analysis.

### 5-ethynyl-20-deoxyuridine (EdU) staining proliferation assay

The proliferation of CC cells was measured by using EdU assay kit (RiboBio, Guangzhou, China). In breif, the transfected CC cells were incubated with 50 μm EdU for 2 h. And the cell nuclei were dyed with 4', 6-diamidino-2phenylindole (DAPI, Sigma, USA). Finnally, the EdU-positive cells were observed by using a fluorescence microscope.

### Cell cycle

Pre-chilled 1 X PBS used to re-suspended cells. 1× Annexin binding buffer diluted cells to 1×10^5^ cells/ml. Cells were precooled with 70% ethanol at 4 °C overnight before assay.

### Flow cytometry

Transfected cells were dyed by Annexin V-FITC Apoptosis Detection Kits (BD, USA). Then collected and washed using phosphate-buffered saline (PBS). Added to 1× binding buffer and resuspended cells, then mixed into the test container 500 μL cell suspension, 5 μL Annexin V-FITC, and 5 μL propidium iodide solution incubated for 15 minutes. The samples apoptosis was detected by a FACScan flow cytometer analyzer (BD, USA).

### Western blot analysis

Proteinase inhibitor (Solarbio) was added and tissue lysate was extracted using RIPA reagent. The concentration of protein was measured by BCA reagent (Beyotime Institute of Biotechnology). Then proteins were separated by 10% SDS-PAGE and transferred to PVDF membrane (Millipore, USA). After incubation with 5% skimmed milk in TBST, PBS was used to washing membrane and then cultivated with the primary antibody overnight at 4°C. This study used following antibodies: anti-TLN1 antibody (1:1000, Abcam, USA) and anti-β-actin antibody (1:1000, Santa Cruz, USA). After that we used the individual antibody for western blot analysis. The signals were observed by an enhanced chemiluminescence detection reagent (GE Healthcare, USA).

### Dual luciferase reporter assays

CircRNA_400029 and miR-1285-3p were predicted having the binding sequences by RegRNA 2.0 16. Similarly, TLN1 and miR-1285-3p were also predicted having the binding sequences by TargetScan database 17. Next to verify whether the circRNA_400029 and TLN1 as a directly target of miR-744-5p, then the wild type circRNA_400029 sequences (circRNA_400029-wt), mutant circRNA_400029 sequences (circRNA_400029-mut), wild type TLN1 3'UTR sequences (TLN1 3'UTR-wt) and mutant TLN1 3'UTR sequences (TLN1 3'UTR-mut) were cloned into the pGL-3 luciferase reporter vector (Promega, USA). The reporter vectors with miR-744-5p or miR-NC were constructed into 293T cells by Lipofectamine 2000 (Invitrogen, USA). After transfection for 36 h we detected the activity by Dual Luciferase Reporter Assay System (Promega, USA).

### Tumor xenograft in nude mice

Obtained four/five-week-old female BALB/c nude mice from Beijing Vital River Laboratory Animal Co., Ltd., (Beijing, China). Resuspend the cell concentration in PBS to 1×10^7^ cells/ml. The cell suspension was then injected subcutaneously into the flanks of nude mice (0.1 ml). Measure the width and length of the tumor every 3 days. All nude mice were sacrificed three weeks after injection, xenogeneic tumors were excised and weighed. The tumor volume is calculated as (length×width^2^)/2. The animal experiment procedure was approved by the Animal Care and Use Committee of Liaoning Cancer Hospital.

### Statistical analysis

All data were displayed as mean ± standard deviation (SD) of more than 3 experiment repeats and statistical analyses were finished by SPSS 23.0 software. The significance differences were analyzed by Student's *t*-test or one-way analysis of variance (ANOVA), *P*<0.05 considered as statistical significance.

## Results

### CircRNA_400029 was upregulated in cervical cancer cells and tissues

**Figure 1:**
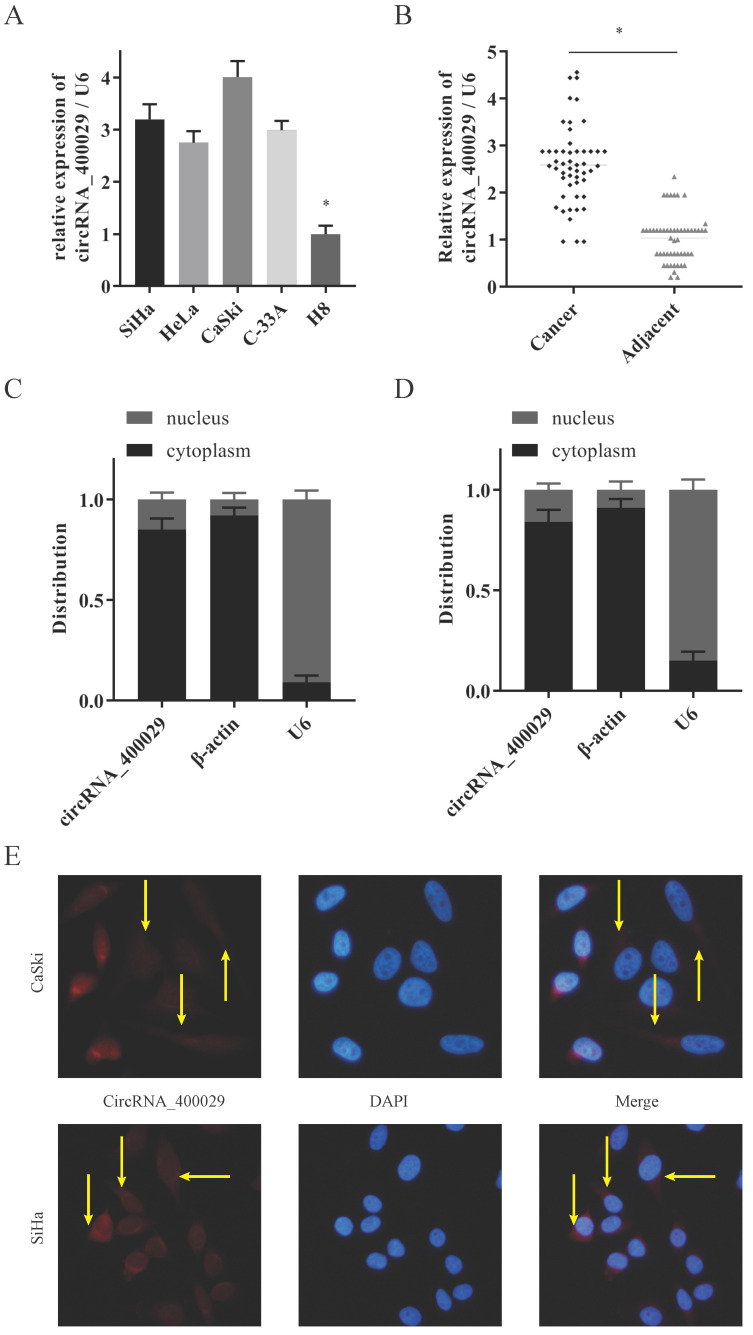
** CircRNA_400029 was overexpressed in cervical cancer. A.** CircRNA_400029 was overexpressed in CC cells and tissue. **B.** CircRNA_400029 expression in CC tissue compare to paired adjacent tissue in 50 patients. **C, D.** The expression level of circRNA_400029 in the subcellular fractions of CC cells was detected by RT-PCR. U6 and β-actin were used as nuclear and cytoplasmic markers, respectively. **E.** Immunofluorescence indicated that CircRNA_400029 was mainly localized in the cytoplasm (Yellow arrows). U6 was used as a loading control in RT-PCR; n=3, *P< 0.05.

To investigate the potential role of circRNA_400029 in CC, we firstly detected the RNA level of circRNA_400029 in CC cells and tissues. RT-PCR showed that circRNA_400029 was significantly increased in CC cells, especially in SiHa and CaSki (Fig. 1A). Besides, circRNA_400029 was also up-regulated in CC tissues compared with adjacent normal tissues (Fig. 1B). These results indicated that circRNA_400029 might play an oncogenic role in CC. Subsequently, we tested the localization of circRNA_400029 in cells in order to further explore the function of circRNA_400029 in CC. The nuclear/cytoplasmic RNA fractionation in the subcellular distribution assay exhibited that circRNA_400029 was mainly located at cytoplasmic in both SiHa (Fig. 1C) and CaSki (Fig. 1D). Not surprisingly, the immunofluorescence also showed the similar results (Fig. 1E). So, we speculated that circRNA_400029 might participate in the corresponding molecular regulation and function at the post-transcriptional level. In addition, we chose the median of circRNA_400029 expression in CC samples as the cutoff value. The CC samples were classified as high- and low- groups based on the expression of circRNA_400029. We found that the expression of circRNA_400029 was closely related to histology, tumor differentiation, and TNM stage (Table 1).

### MiR-1285-3p targeted circRNA_400029 and negative regulated the expression of circRNA_400029

**Figure 2:**
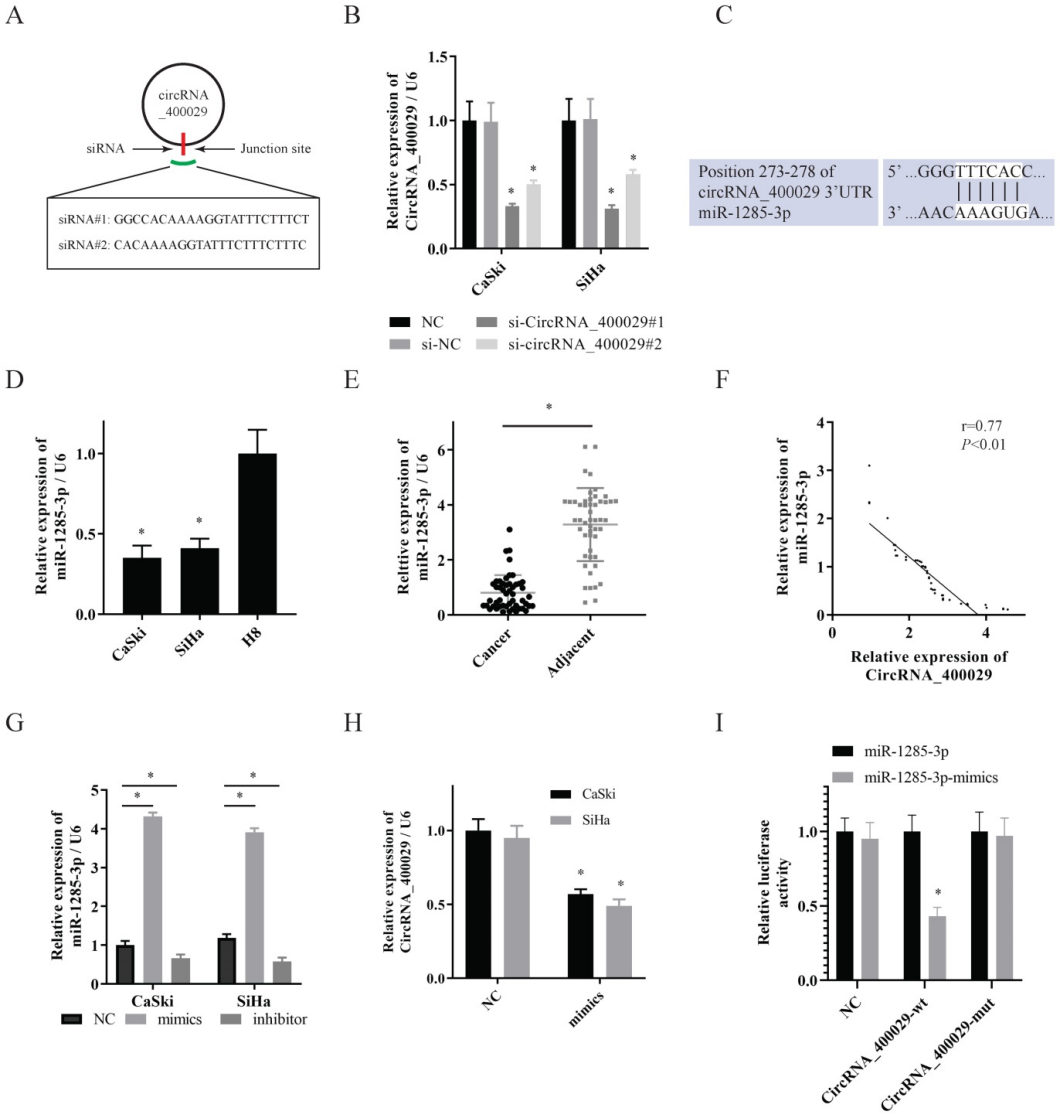
** CircRNA_400029 could crosstalk with miR-1285-3p through direct binding. A.** The diagram showed designed siRNAs targeting the junction site of circRNA_400029. **B.** RT-PCR analysis verifying the knockdown efficiency of siRNAs. **C.** The potential binding sites of circRNA_400029 and miR-1285-3p transcript. **D,** E. miR-1285-3p was weak-expressed in both CC cells and tissue. **F.** The expressions of circRNA_400029 and miR-1285-3p were negatively correlated. **G.** RT-PCR analyzed miR-1285-3p expression in CC cells treated with mimics and inhibitor. **H.** The mimics of miR-1285-3p inhibited the expression of circRNA_400029. **I.** Luciferase activity in 293T co-transfected with luciferase reporter encompassing circRNA_400029 and miR-1285-3p mimics. Results represent renilla and firefly luciferase activity ratio.

Then two siRNAs sequences were designed for circRNA_400029 junction sites to inhibit circRNA_400029 expression (Fig. 2A). Next, we transfected SiHa and CaSki cells with circRNA_400029 siRNAs. Among them, siRNA- circRNA_400029#1 had a better silence efficiency (Fig. 2B). CircRNA_400029 was predicted containing the binding site of miR-1285-3p by RegRNA 2.0 (Fig. 2C). Moreover, the expression of miR-1285-3p in CC cells and tissues was explored via RT-PCR. As expected, miR-1285-3p was weak-expressed in cells and tissues (Fig. 2D, 2E). The RT-PCR observations indicated that a negatively regulative association between circRNA_400029 and miR-1285-3p (r=0.77, *P*<0.01, Fig. 2F). Next, the mimics and the inhibitor of miR-1285-3p were transfected into CC cells to knockdown or up-regulated the expression of miR-1285-3p (Fig. 2G). Interestingly, the expression level of circRNA_400029 was detected in CC cells transfected with mimics of miR-1285-3p and the results showed that up-regulation of miR-1285-3p significantly inhibited the expression of circRNA_400029 (Fig. 2H). To confirm that, we constructed the pGL3 luciferase reporter plasmid containing the wild type circRNA_400029 sequences (circRNA_400029-wt) and mutant circRNA_400029 sequences (circRNA_400029-mut). Dual luciferase reporter assay revealed that miR-1285-3p decreased the luciferase expression of circRNA_400029-wt in 293T cells whereas the circRNA_400029-mut was not affected (Fig. 2I).

### CircRNA_400029 functioned as ceRNA of TLN1 via sponging miR-1285-3p

**Figure 3:**
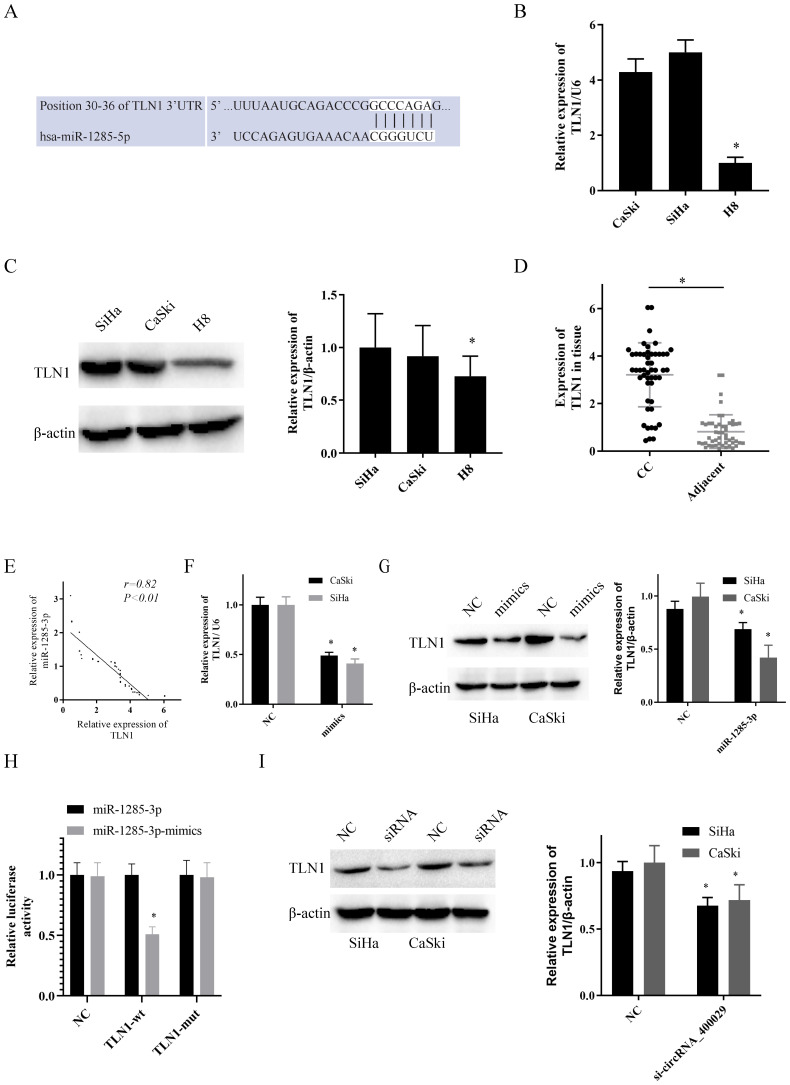
** TLN1 was overexpressed in CC and identified as a target of miR-1285-3p. A.** Predicted binding sites for miR-1285-3p on the TLN1 transcript. **B, C.** TLN1was overexpressed in CC cells compare to it in H8 by RT-PCR and western-blot. **D.** TLN1 was overexpressed in CC tissue. **E.** TLN1 was negatively correlated with the expression of miR-1285-3p. **F, G.** CC cells were transfected with the mimics of miR-1285-3p Reduced TLN1 expression was shown by RT-RCR and western-blot. **H.** Luciferase activities were measured in 293T cells co-transfected with luciferase reporter containing TLN1 and the mimics of miR-1285-3p. **I.** The expression of TLN1 was decreased with the knocked down of circRNA_400029. Data are shown as mean ± SD, n = 3. The data statistical significance is assessed by Student's t-test. *P < 0.05.

To uncover the potential target genes mediated by miR-1285-3p in CC cell, we identified TLN1 as one of the target gene of miR-1285-3p through the bioinformatics TargetScan database (Fig. 3A). The RT-PCR and western-blotting observations showed a substantial increase of TLN1 was observed in CC cell lines than in H8 (Fig. 3B, 3C). Subsequently, we also detected the expression of TLN1 CC and adjacent tissues. Consistent with circRNA_400029, TLN1 was also overexpressed in CC (Fig. 3D) and negatively correlated with miR-1285-3p expression (r=0.85, *P*<0.01, Fig. 3E). In addition, over-expressed miR-1285-3p could inhibit TLN1 mRNA and protein level (Fig. 3F, 3G). Based on this result, Dual-luciferase reporter assays observed that miR-1285-3p mimics transfection distinctly declining the luciferase signal of reporters comprising TLN1-WT, but no impact was detected on the luciferase signal of reporters comprising TLN1-MUT (Fig. 3H). The siRNA of circRNA_400029 could inhibited the protein level of TLN1 (Fig. 3I).

### The biological function of circRNA_400029/miR-1285-3p/TLN1 axis *in vitro*

**Figure 4:**
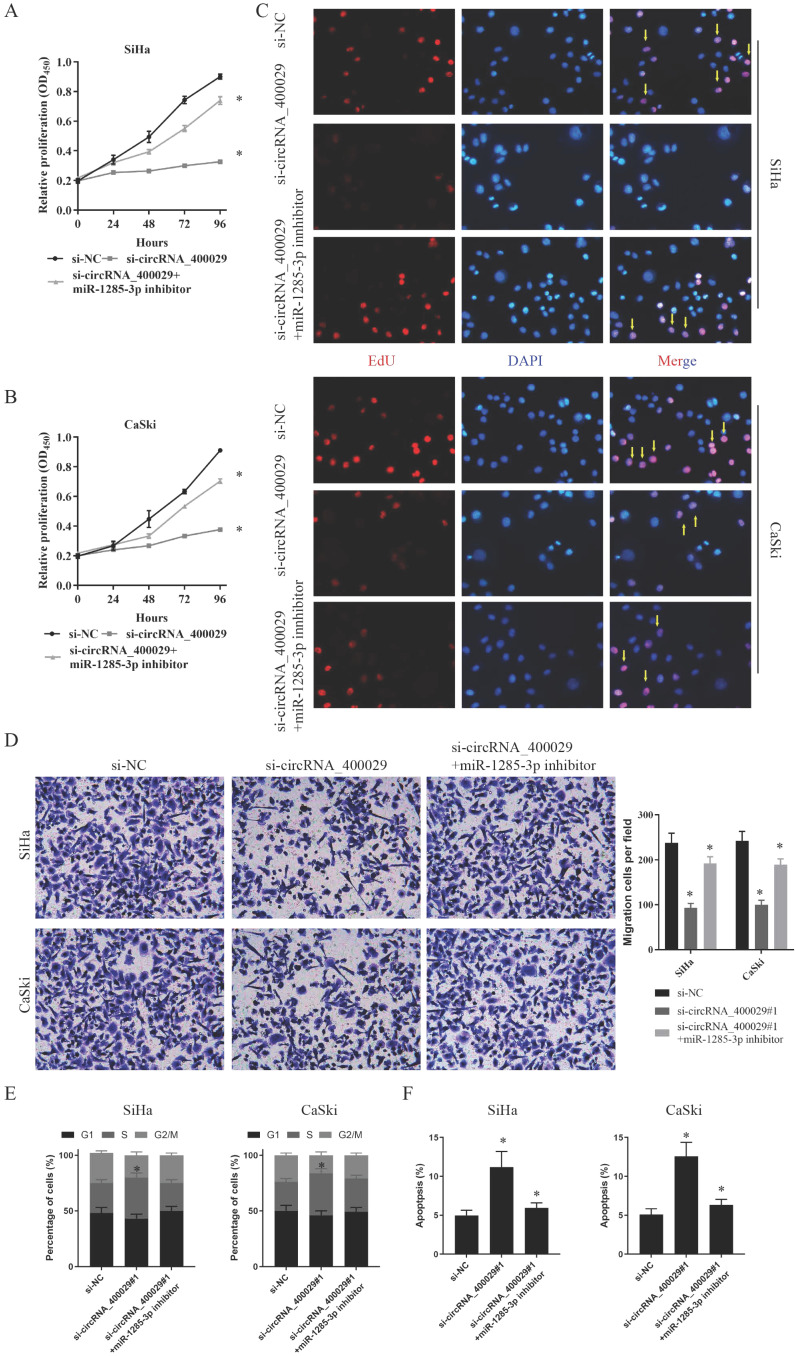
** CircRNA_400029 reinforces the proliferative and invasive. A, B.** Cell proliferation assessed in circRNA_400029 knockdown and circRNA_400029 knockdown + miR-1285-3p inhibitor by CCK-8. A: SiHa; B: CaSki. **C.** Cell proliferation assessed in circRNA_400029 knockdown and circRNA_400029 knockdown + miR-1285-3p inhibitor by Edu. up: SiHa; down: CaSki (si-NC contained more merge cells than si-circRNA_400029 and si-circRNA_400029 + miR-1285-3p inhibitor. si-circRNA_400029 + miR-1285-3p inhibitor contained more merge cell than si-circRNA_400029, marked with yellow arrows). **D.** Transwell assays were used to evaluate the involvement of circRNA_400029 for invasion in circRNA_400029 knockdown and circRNA_400029 knockdown + miR-1285-3p inhibitor. up: SiHa; down: CaSki. **E.** The cell cycle progression of CC cells transfected with si-NC, si-circRNA_400029, si-circRNA_400029 + miR-1285-3p inhibitor was identified by flow cytometry assay. **F.** Effects of si-circRNA_400029, si-circRNA_400029 + miR-1285-3p inhibitor on CC cell apoptosis. Data are shown as mean ± SD, n = 3. The data statistical significance is assessed by Student's* t*-test. *P < 0.05.

To further explore the functional effects of circRNA_400029 in CC cell lines, we chose the SiHa and CaSki cell lines for further phenotype experiments. Firstly, CCK-8 assay indicated that proliferation of CC cells transfected with si-circRNA_400029 was significant inhibited. On this basis, both si-circRNA_400029 and the inhibitor of miR-1285-3p were transfected; the cell proliferation ability was restored. It suggested that the effect of circRNA_400029 on CC proliferation was realized through miR-1285-3p (Fig. 4A, 4B). We conducted an EdU staining to further verify this conjecture. As expected, the fluorescence intensity of CC cells decreased after circRNA_400029 knockdown. The fluorescence intensity of CC cells recovered after siRNA and inhibitor co-transfection. This further supports the role of circRNA_400029 in CC cell proliferation (Fig. 4C). Moreover, the migration and invasion of SiHa and CaSki cells transfected with si-circRNA_400029 or miR-1285-3p inhibitor were detected by Transwell assay. The results revealed that down-regulation of circRNA_400029 suppressed CC cells migration and invasion (Fig. 4D). Furthermore, flow cytometry assessed cell cycle progression. Results confirmed that si-circRNA_400029 significantly increased the proportion of CC cells in S phase but decreased the proportion in G2/M phase. However, it could be recovered by miR-1285-3p inhibitor (Fig. 4E). Nevertheless, flow cytometry assay showed that transfection of si-circRNA_400029 result in an obvious increase of apoptosis rates in both SiHa and CaSki cells. And with the decreased of miR-1285-3p expression, apoptosis decreased (Fig. 4F).

**Figure 5:**
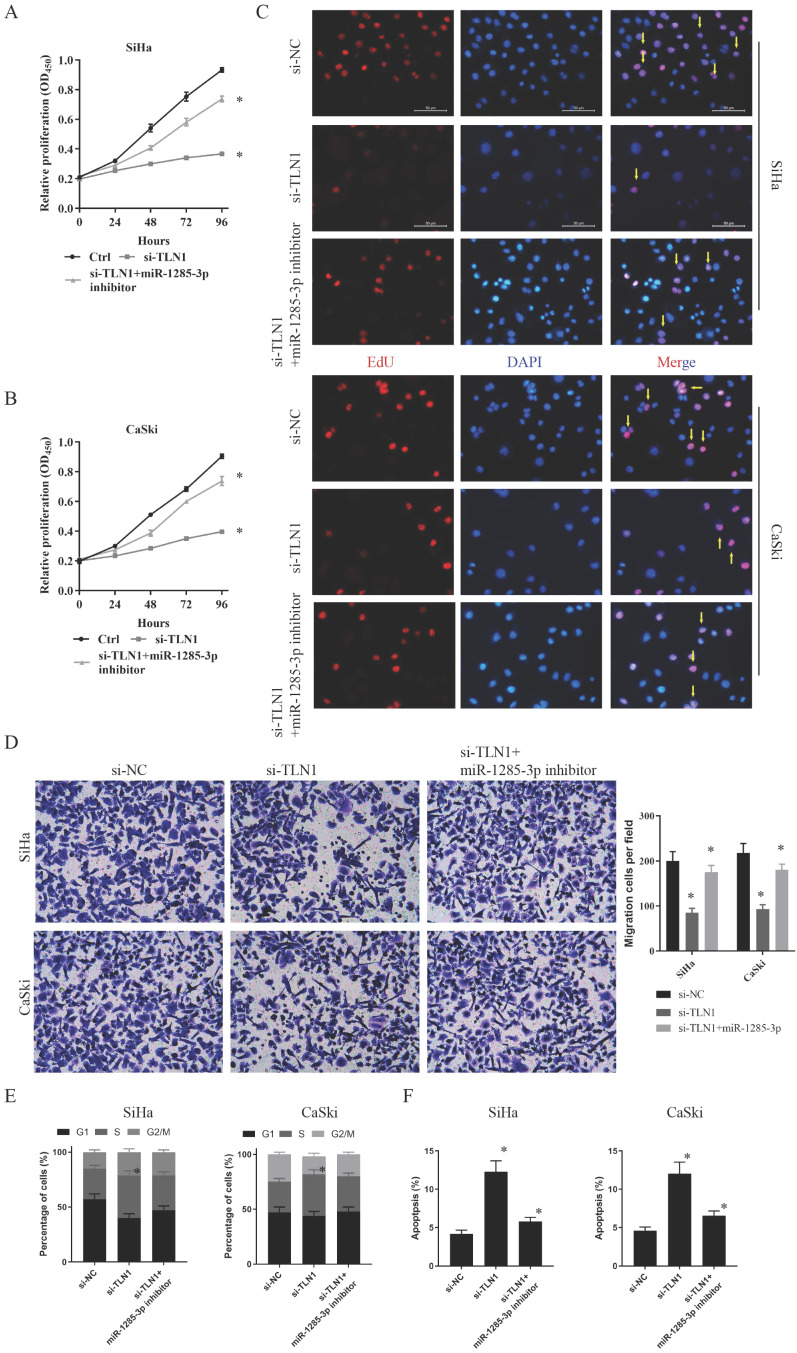
** TLN1 could crosstalk with miR-1285-3p through direct binding. A, B.** Cell proliferation assessed in TLN1 knockdown and TLN1 knockdown + miR-1285-3p inhibitor by CCK-8. A: SiHa; B: CaSki. **C.** Cell proliferation assessed in TLN1 knockdown and TLN1 knockdown + miR-1285-3p inhibitor by Edu. up: SiHa; down: CaSki (si-NC contained more merge cells than si-TLN1 and si-TLN1 + miR-1285-3p inhibitor. si-TLN1 + miR-1285-3p inhibitor contained more merge cell than si-TLN1, marked with yellow arrows). **D.** Transwell assays were used to evaluate the involvement of TLN1 for invasion in TLN1 knockdown and TLN1 knockdown + miR-1285-3p inhibitor. up: SiHa; down: CaSki. **E.** The cell cycle progression of CC cells transfected with si-NC, si-TLN1, si-TLN1 + miR-1285-3p inhibitor was identified by flow cytometry assay. **F.** Effects of si-TLN1, si-TLN1 + miR-1285-3p inhibitor on CC cell apoptosis. Data are shown as mean ± SD, n = 3. The data statistical significance is assessed by Student's* t*-test. *P < 0.05.

Furthermore, the effects of miR-1285-3p and TLN1 on CC proliferation and migration were verified by the same method. CCK-8 assays displayed that an apparently reduced cell viability in si-TLN1 group, while a substantially augmented cell viability in si-TLN1 + miR-1285-3p inhibitor group (Fig. 5A, 5B). Similarly, the knockdown of TLN1 also reduced fluorescence intensity of CC cells. With the decrease of miR-1285-3p expression, the fluorescence intensity of the cells recovered (Fig. 5C). Moreover, the migration or invasion ability of CC cells were restrained when carrying si-TLN1 transfection, whereas strengthen when carrying both si-TLN1 and miR-1285-3p inhibitor transfection (Fig. 5D).

Similarly, we also performed flow cytometry assessed to evaluate cell cycle progression and apoptosis. Results indicated that si-TLN1 increased S phase cells, but decreased G2/M phase proportion. And miR-1285-3p inhibitor could reverse this phenomenon (Fig. 5E). Besides, si-TLN1could induce cell apoptosis. And with the decreased of miR-1285-3p expression, apoptosis decreased, too (Fig. 5F).

These data suggested that, as competitive endogenous RNA (ceRNA), circRNA_400029 was combined with miR-1285-3p to regulate the expression of TLN1, thus affecting CC cells proliferation, migration and invasion.

### The biological function of circRNA_400029/miR-1285-3p/TLN1 axis *in vivo*

To clarify the biological functions of circRNA_400029/miR-1285-3p/TLN1 axis *in vivo*. Different SiHa cells were subcutaneously injected into nude mice.

**Figure 6:**
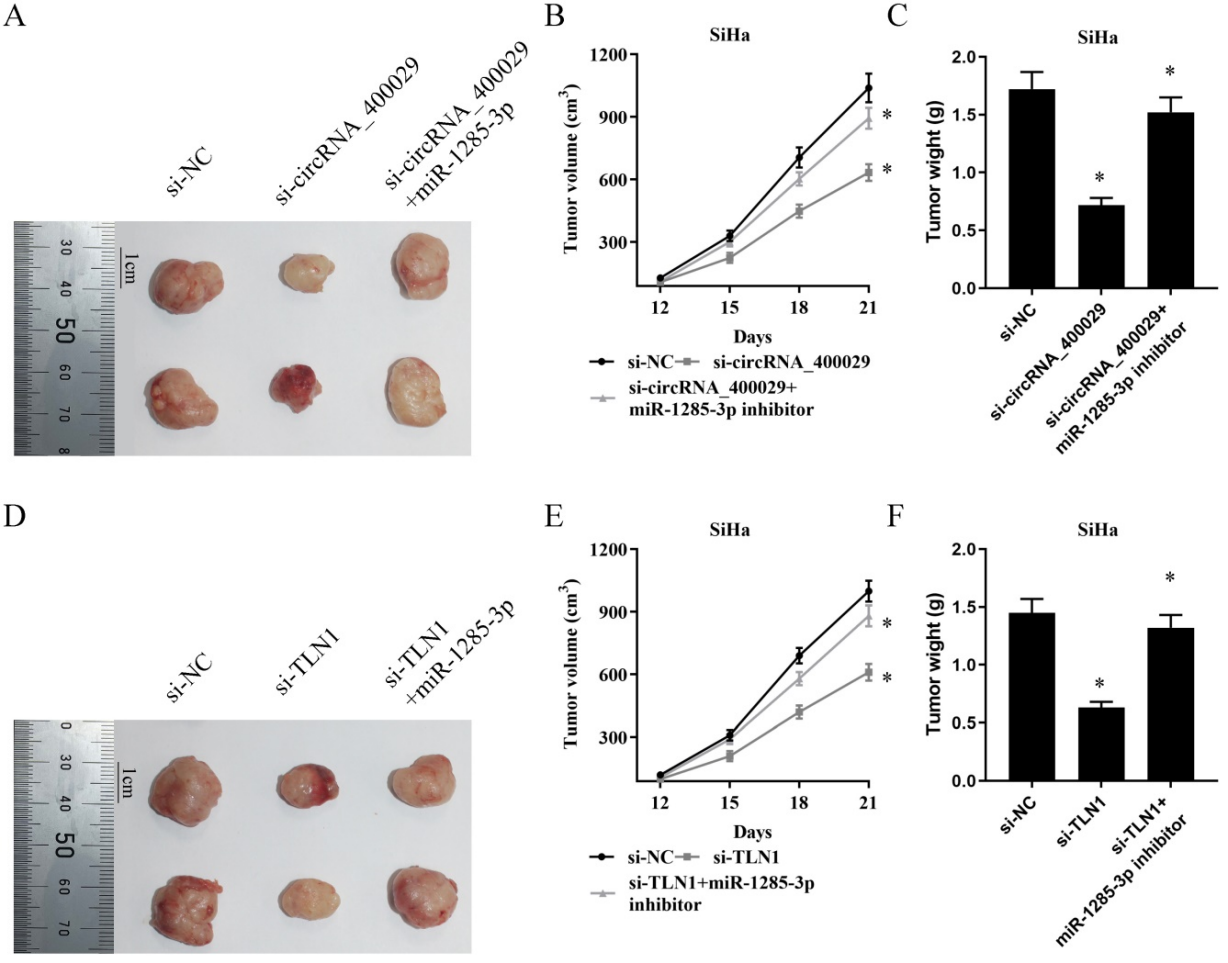
***In vivo* tumor lumps of xenograft mouse models composed of circRNA_400029, miR-1285-3p and TLN1. A.** Representative images of the tumor lumps of each group at the endpoint of the experiment. **B.** The tumor growth curves of *in vivo* tumor volumes. **C.** The mean tumor weight of each group. Data are mean ± SD. of the tumor volumes, n = 5, *P < 0.05.

In total, there were six groups: Group 1. si-NC was injected with SiHa cells; Group 2. si-circRNA_400029 was injected with SiHa cells; Group 3. si-circRNA_400029 + miR-1285-3p inhibitor was injected with SiHa cells; Group 4. si-NC was injected with SiHa cells; Group 5. si-TLN1 was injected with SiHa cells; Group 6. si-TLN1 + miR-1285-3p inhibitor was injected with SiHa cells (n = 5 per group). We measured the tumor volumes every 3 days. Moreover, the tumor volumes in the si-circRNA_400029 and si-TLN1 groups were smaller than in si-NC group. The mice were sacrificed at the end of the experiment, and the tumor volume and weight of each group were measured (Fig. 6A, 6D).

The mean tumor volume at the time of death in mice injected with si-circRNA_400029 cells was 633.21 ± 41.00 (mean value ± SD) mm^3^ and the mean tumor volume of mice injected with si-NC cells was 1038.91 ± 69.47 mm^3^. And the inhibitor of miR-1285-3p could reverse this phenomenon. The volume of si-circRNA_400029 + miR-1285-3p group of tumors was 893.30 ± 52.61 mm^3^ (Fig. 6B). Similarly, si-circRNA_400029 also limited tumor weight (0.72 ± 0.06 g *vs* si-NC: 1.72 ± 0.15 g), whereas miR-1285-3p inhibitor rescued this condition (1.52 ± 0.13 g, Fig. 6C). We get similar results in TLNN1 and miR-1285-3p: si-TLN1 decreased the volume and weight of tumors (610.81 ± 51.20 mm^3^, 0.63 ± 0.05 g, *vs* si-NC: 999.57 ± 50.29 mm^3^, 1.45 ± 0.12 g, Fig. 6E, 6F). The miR-1285-3p inhibitor could increase the volume and weight of tumors (880.27 ± 50.79 mm^3^, 1.32 ± 0.11 g, Fig. 6E, 6F).

## Discussion

CC was the second most common gynecological malignant, in recent years, its onset age has a tendency to become younger 18. More and more researches have been devoted to elucidating the genetic and epigenetic mechanisms involved in the progression of CC 19, but the pathogenesis of CC is still not fully understood. Current studies have shown that circRNA was considered as a biomarker related to clinical testing and could contribute to the poor prognosis of various tumors due to its stable structure 20. CircRNA as an essential part of the human transcriptome, the type is various 21. Sequence studies confirm circRNA abnormal expression in different cancer by apparent after modification, transcription 22 and its regulation and regulating tumor related gene expression of the tumor cell proliferation and invasion have an vital impact in the process of development 23, 24. Meanwhile, it has been reported that circRNA can be used as an anti-tumor target for the therapy of cancer and has certain clinical application value 25.

CircRNA_400029, also called circ_0092337, was located chr 16: 89628179- 89628539, and derived from ribosomal protein L13 (RPL13). Li *et al* reported that circRNA_400029 could be used as plasma circRNA in molecular detection of gastric cancer 26. Our study confirmed that circRNA_400029 was highly expressed in both cervical cancer tissues and cells. We found and demonstrated that circRNA_400029 could promote CC proliferation, invasion and inhibit apoptosis. It suggested that circRNA_400029 might play an important role in the progression of CC. CircRNA has been reported to induce cervical cancer progression not only by altering chromatin modifications and mRNA stability at the transcriptional level, but also by binding to miRNAs to serve as ceRNA or promoting protein stabilization at post-transcriptional levels 27. To explore whether circRNA_400029 and miRNAs can provide new insights on the mechanisms of CC proliferation and invasion, FISH clearly showed that circRNA_400029 is localized in the cytoplasm, supporting the hypothesis that circRNA_400029 regulates gene expression at the post-transcriptional level through the ceRNA mechanism. Afterwards, we used miRanda and RNAhybrid algorithm to analyze the microarray data. The prediction was combined with the RegRNA2.0 database. We found that miR-1285-3p may be a potential target of circRNA_400029. CircRNA_400029 could inhibit the expression of miR-1285-3p. miR-1285-3p antagonized the effects of circRNA_400029 on the proliferation and invasion of CC cells. The dual luciferase reporter assays confirmed the direct relationship between the two factors. Bioinformatics analysis revealed that miR-1285-3p was a potential tumor suppressor gene for breast cancer 28. Previous reports presented that miR-1285-3p modulated multiple molecular targets and served as a sponge in ovarian cancer 29, oral squamous cell carcinoma 30, and cervical cancer 31. Upregulated the expression of miR-1285-3p also inhibited cell proliferation and escape from apoptosis of colorectal cancer 32. Our study confirmed that the expression level of circRNA_400029 was negatively correlated with miR-1285-3p in CC tissues and cells. Luciferase reporter gene assay confirmed that miR-1285-3p could complement and bind to miR-1285-3p.This suggested that circRNA_400029 acted as a sponge for miR-1285-3p in CC, thus promoting the progression of cervical cancer.

We identified TLN1 as an unreported potential target of miR-1285-3p using an online predictive database. The direct binding relationship between TLN1 and miR-1285-3p was confirmed by luciferase reporter gene assay. In CC cells, TLN1 was positively regulated by circRNA_400029 and negatively regulated by miR-1285-3p -mimic, suggesting that TLN1 was also a target gene of miR-1285-3p regulated by circRNA_400029. TLN1 gene encoded a cytoskeletal protein that was concentrated in areas of cell-substratum and cell-cell contacts. It played a significant role in the assembly of actin filaments and in spreading and migration of various cell types, including fibroblasts and osteoclasts. It codistributed with integrins in the cell surface membrane in order to assist in the attachment of adherent cells to extracellular matrices and of lymphocytes to other cells. Overexpression of TLN1 activated the progression of gastric cance through PTK2-PXN-VCL-E-Cadherin-CAPN2-MAPK1signaling axis 33. Besides this, TLN1 overexpression promoted hepatocellular carcinoma epithelial-mesenchymal transition and induced metastasis 34. In addition, the copy number alterations of TLN1 was contributed the tumorigenesis of neoplasias 35. It was considered as a potential therapeutic target for cancer treatment 36. In this study, we confirmed that circRNA_400029 silencing can inhibit the proliferation, metastasis and invasion ability of CC cells, as well as inhibit the change of cell cycle, and miR-1285-3p inhibitor can eliminate the biological function changes mediated by circRNA_400029 silencing. Consistent with *in vitro* results, down-regulation of circRNA_400029 inhibited xenograft growth in mice *in vivo*. Meanwhile, TLN1 interference can reverse circRNA_400029 mediated changes in cell activity, and TLN1 interference combined with miR-1285-3p inhibitor can enhance this reversal effect.

In conclusion, our study revealed that circRNA_400029 plays a positive role in regulating the growth of cervical cancer. The underlying mechanism is circRNA_400029 as a competitive endogenous RNA, through circRNA_400029/ miR-1285-3p/ TLN1 axis regulates the expression of TLN1, further affecting tumor growth, invasion and metastasis and changing cell cycle. These results provide a new idea for the mechanism between circRNAs and cervical cancer.

## Figures and Tables

**Table 1:** CircRNA_400029 expressions and clinicopathologic characteristics

Characteristics	circRNA_400029		*P*	X^2^
	Low (%)	High (%)		
	26	24		
**Age**			0.59	0.31
≥45	12	11		
<45	14	13		
**Histology**			0.00	12.86
Adenocarcinoma	3	10		
Squamous carcinoma	23	14		
**Tumor Size**			0.36	0.93
≥4 cm	13	10		
<4 cm	13	14		
**HPV infection**			0.72	0.40
Negative	2	1		
Positive	24	23		
**Tumor differentiation**			0.00	10.05
Moderate and high	11	19		
Poor	15	5		
**Lymph node invasion**			0.17	1.36
Yes	10	11		
No	16	13		
**TNM stage**			0.03	5.59
I	11	6		
II	15	18		
